# Autoimmune encephalitis followed by hemophagocytic lymph histiocytosis: a case report

**DOI:** 10.3389/fimmu.2024.1383255

**Published:** 2024-07-22

**Authors:** Li Huang, Jie Tan, Peihao Lin, Zixuan Chen, Qihua Huang, Haiyan Yao, Lihong Jiang, Baoyi Long, Youming Long

**Affiliations:** ^1^ Department of Neurology, The Second Affiliated Hospital of Guangzhou Medical University, Guangzhou, China; ^2^ Key Laboratory of Neurogenetics and Channelopathies of Guangdong Province and The Ministry of Education of China, Institute of Neuroscience and the Second Affiliated Hospital of Guangzhou Medical University, Guangzhou, China; ^3^ Second Clinical College, Guangzhou Medical University, Guangzhou, China; ^4^ First Clinical College, Changsha Medical University, Changsha, Hunan, China

**Keywords:** autoimmune encephalitis, hemophagocytic lymphohistiocytosis, autoantibody, lymphoma, paraneoplastic neurologic syndromes

## Abstract

**Objective:**

This study aims to report three cases of autoimmune encephalitis followed by hemophagocytic lymphohistiocytosis.

**Methods:**

Data of relevant patients treated between 2019 and 2022 were retrospectively collected from the Department of Neurology at the Second Affiliated Hospital of Guangzhou Medical University.

**Results:**

The age at onset of the three patients was 37, 63, and 36 years, respectively. All three patients were female and presented with cognitive dysfunction and seizures. Behavioral and psychological symptoms were also observed in two cases. All patients were positive for autoantibodies in both the cerebrospinal fluid and serum, while two showed multiple abnormal brain signals on magnetic resonance imaging. All patients exhibited hypocytosis and elevated soluble CD25 and serum ferritin levels. The final diagnoses in two cases were lymphomas, while the remaining case without tumors suffered from a severe infection. All patients received immunotherapy, and the two with lymphoma received anti-tumor treatment. The patient with infection died, and two patients with tumors improved after chemotherapy.

**Conclusion:**

Autoimmune encephalitis followed by hemophagocytic lymphohistiocytosis is a rare and severe condition. Prompt attention should be paid to the decline in blood cell counts, particularly in patients who show a slight improvement after immunotherapy or have a risk of lymphoma. Screening for potential tumors and infections and early treatment may help these patients.

## 1 Introduction

Autoimmune encephalitis (AE) is a form of neurological dysfunction caused by diffuse or multiple inflammatory lesions mediated by autoimmune mechanisms in the brain parenchyma. AE is the most frequent type of encephalitis, with prior research showing that it has a higher incidence than viral encephalitis (1). Some cases of antibody-positive AE are closely related to the underlying tumor and present as paraneoplastic neurological syndromes (PNS) (2). Hemophagocytic syndrome, also known as hemophagocytic lymphohistiocytosis (HLH), is a disease characterized by immune hyperactivation and dysregulation, which can be caused by either infections or tumors, with a variety of clinical manifestations and a resulting high incidence of delayed diagnosis (3). Associations between AE and HLH have been reported in rare cases. To the best of our knowledge, only one case of anti-N-methyl-D-aspartate receptor (NMDAR) AE followed by HLH has been reported, and this patient improved after immunotherapy without chemotherapy (4).

From 2020 to 2022, we encountered three cases of AE followed by HLH in our center. The three cases all initially improved after immunotherapy; however, they worsened and were found to have HLH at 20, 36, and 90 days after the initial diagnosis of AE. Of these, two improved after chemotherapy, while one died. In a prior study, Kwak et al. reviewed 2,200 adult HLH cases and concluded that HLH patients with autoimmune diseases or tumors had a poor prognosis and mostly required admission to intensive care units (5). Therefore, clinicians should focus on these conditions. In the present study, we retrospectively analyzed the clinical data of these aforementioned patients and explored the relationship between AE and HLH to share our findings with clinicians.

## 2 Presentation of cases

### 2.1 Case 1

After exercising, a 37-year-old woman developed a debilitating headache at the top of her head, which lasted for tens of minutes and was accompanied by several episodes of non-projectile vomiting. At 4 days later, the headache recurred, accompanied by dizziness and vomiting, but resolved after symptomatic treatment at a local hospital. Thereafter, she experienced intermittent dizziness and drowsiness and was prone to emotional tension. A month later, as headache worsened and she experienced fever reaching a peak of 38.5°C, she was admitted to our hospital. Paroxysmal babbling and crying occurred twice, and her reaction worsened. A physical examination revealed a temperature (T) of 36.6°C; heart rate (P), <82 times/min; respiration (R) 18 times/min; and blood pressure (BP), 126/83 mmHg. She was further found to have decreased memory and numeracy and positive bilateral Rossolimo signs. Her neck was slightly stiff, and the chin–chest distance was between two transverse fingers.

**Figure 1:**
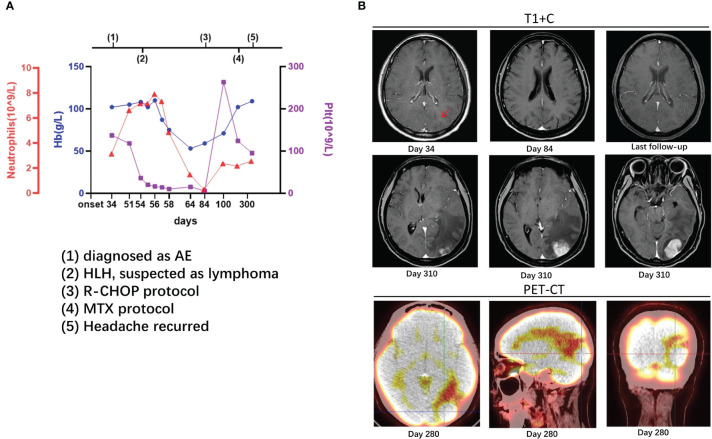
Case 1: A 37-year-old woman experienced hemophagocytic lymphohistiocytosis 20 days after her diagnosis of autoimmune encephalitis. A 37-year-old lady was admitted to our hospital with headache and vomiting for 1 month. Her serum and cerebrospinal fluid were positive for anti-neuron antibodies. On enhanced MRI, there were high signals in the cortex of the left occipital lobe [**(B)**, day 34, arrow] and enlarged cerebral sulcus vessels. She was diagnosed as a case of autoimmune encephalitis. On the 54th day, her platelet count suddenly dropped, and other examinations, such as bone marrow biopsy, supported lymphoma-associated hemophagocytic lymphohistiocytosis **(A)**. On the 84th day, the enhancement remained in the cortex of the left occipital lobe [**(B)**, day 84]. The counts of platelet, hemoglobin, and neutrophils recovered after the administration of R-CHOP protocol **(A)**. On the 280th day, lesions with high metabolism in the left occipital lobe were found on PET-CT, and methotrexate was administrated [**(B)**, day 280], but headache recurred a month later [**(B)**, day 310]. After MTX protocol and autologous stem cell transplantation, the lesions reduced without new ones on imaging at follow-up [**(B)**, last follow-up]. The details are discussed in the main text.

The patient scored 23 on the Mini-Mental State Examination (MMSE) and 22 on the Montreal Cognitive Assessment (MoCA). The head MRI with T2-weighted imaging (T2WI), fluid-attenuated inversion recovery (FLAIR), and enhancement sequencing revealed a high signal intensity in the cortex of the left occipital lobe and enlarged cerebral sulcus vessels (**Figure 1**). There were no serological abnormalities. The cerebrospinal fluid (CSF) was colorless and clear, with 1,313 mg/L protein (range: 150–400 mg/L) and 16 × 10^6^/L white blood cells (WBC, range: 0–5 ×10^6^/L). The CSF shared oligoclonal bands with the serum (type IV). Next-generation sequencing testing of the CSF was negative. A tissue-based assay (TBA) showed that both the serum and CSF were positive for anti-neuron antibodies, while anti-NMDAR, anti-AMPA1, anti-AMPA2, anti-LGI1, anti-GABAB, and anti-CASPR2 antibodies were negative in the cell-based assay (CBA). Visual-evoked potentials (VEP) showed conduction delays in the bilateral visual pathways, and electroencephalography (EEG) showed increased energy in the alpha band in all leads, which was evident in the occipital region and mainly slow waves. Therefore, the patient was diagnosed with AE, which improved after steroid and immunoglobulin treatments.

On the 54th day after onset, the patient suddenly developed babbling with decreased consciousness. Considering delirium, olanzapine and phenobarbital were administered but without significant improvement. A serological examination revealed the following: 3.35 × 10^12^/L of red blood cells (RBC, range: 4.00–5.00 × 10^12^/L), 108 g/L of hemoglobin (Hb, range: 110–150 g/L), 28 × 10^9^/L of platelet (Plt, range: 125–320 × 10^9^/L), 18,243 U/mL of soluble interleukin 2 receptor (sCD25, range: 223–710 U/mL), 3,799.16 ng/mL of serum ferritin (range: 4.63–204.00 ng/mL), and positive Epstein–Barr virus (EBV) DNA. The fluctuations in hemoglobin, platelets, and neutrophils are shown in **Figure 1**. A bone marrow biopsy revealed the proliferation of bone marrow cells, hemophagocytosis, and a large number of heterogeneous lymphocytes infiltrating the interstitium. The results of immunohistochemistry were listed as follows: CD20, CD79a, and MUM1 (+), MPO (myeloid cells +), CD71 (erythroid cells +), CD61 (megakaryocytes +), CD117 (scattered cells weakly positive), CD34 (-), CD3 (few T cells +), BCL2, BCL6, CD10, and CD21 all (-), and Ki-67 (approximately 80% +). Decreased natural killer (NK) cell activity was detected on bone marrow flow cytometry. As such, the patient was diagnosed as having stage IVB diffuse large B-cell lymphoma and lymphoma-associated HLH.

The HLH-2004 protocol of etoposide and dexamethasone was administered for a month, after which treatment was switched to the standard R-CHOP (rituximab plus cyclophosphamide, doxorubicin, vincristine, and prednisone) protocol. During the 7-month R-CHOP administration, her symptoms improved, with no significant changes on head MRI (**Figure 1**). On the 7th month, lymphoma was found on PET-CT, and methotrexate was administrated. However, a month later, the headache recurred, and the lesion in the left occipital lobe was found to be enlarged (**Figure 1**). Therefore, Bruton’s tyrosine kinase inhibitor (BTK), rituximab, pirarubicin (THP), and cytarabine (Ara-C) were added, and autologous stem cell transplantation was performed. At 1 month later, the lesions decreased on computed tomography (CT), and the edema around the lesions significantly diminished. To date (follow-up for more than 2 years), the patient has improved well, without new lesions on imaging.

### 2.2 Case 2

A 63-year-old woman presented with episodic left-sided facial convulsions, salivation from the corner of the mouth, and unresponsiveness without any apparent trigger. She experienced seizures four to five times a day lasting approximately 5 to 6 min, without limb jerking or incontinence. After the seizures, she regained consciousness, but the process could not be recalled. Head CT revealed a low-density lesion in the right temporal lobe, and antiepileptic treatment was administered; however, there was a slight improvement. Positron emission tomography (PET)-CT performed at the local hospital (data unavailable) showed multiple nodules with diminished glucose metabolism in the bilateral temporal lobes, with predominance in the right lobe. Patches of low-density edema were observed around the lesions. The patient visited our hospital on the 10th day.

**Figure 2:**
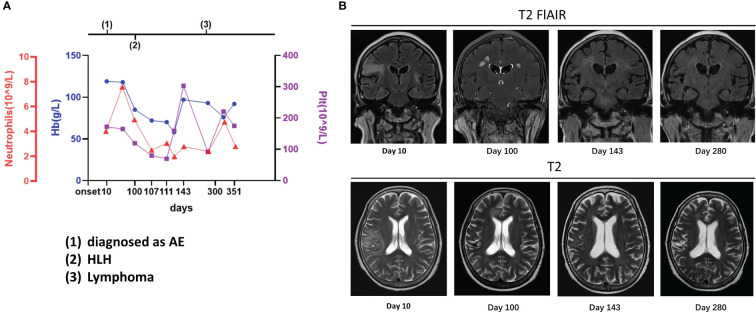
Case 2: A 63-year-old woman experienced hemophagocytic lymphohistiocytosis 90 days after her diagnosis of autoimmune encephalitis. A 63-year-old woman was admitted to our hospital with paroxysmal facial convulsions for 10 days. Her serum and cerebrospinal fluid were positive for anti-CASPR2. The MRI result showed multiple abnormal signals in the bilateral temporal insula [**(B)**, day 10]. On the 100th day, the symptoms recurred, and her counts of platelet, hemoglobin, and neutrophils dropped and the other examinations such as bone marrow biopsy supported hemophagocytic lymphohistiocytosis **(A)**. On MRI, the previous lesions shrank with old hemorrhagic foci, but new abnormal signals were found in bilateral paracingulate gyrus [**(B)**, day 100]. The lymphoma was finally confirmed 6 months later, but the lesions had decreased with foci of malacia, and no new lesions were found on MRI [**(B)**, day 280]. The details are discussed in the main text.

On physical examination, she was found to have diminished pharyngeal reflexes, left-sided sensation, and bilateral heel-to-knee and finger-nose tests. The patient scored 25 on the Mini-Mental State Examination (MMSE) and 20 on the Montreal Cognitive Assessment (MoCA). Electroencephalography (EEG) showed irregular medium-amplitude theta activity in the background, with intermittent single prominent background spike waves. The MRI results showed multiple abnormal signals in the temporal insula bilaterally (**Figure 2**). Serological analysis revealed the following: Hb, 119 g/L; RBC, 3.36 × 10^12^/L; Plt, 172 × 10^9^/L; lactate dehydrogenase, (LDH) 473 U/L (range: 120–250 U/L), and adenosine deaminase (ADA), 42.3 U/L (range: 4–22 U/L). Fluctuations in hemoglobin, platelets, and neutrophils are shown in **Figure 2**. The tumor biomarkers, including neuron-specific enolase (NSE), alpha-fetoprotein (AFP), cancer antigen 19-9 (CA19–9), and cytokeratin 19 fragment (Cyfra21–1), were all negative. The CSF was colorless and clear, with 4 × 10^6^/L of WBC and 623 mg/L of proteins. The CSF shared oligoclonal bands with the serum (type IV). Anti-CASPR2 was positive (1:30) in both the CSF and serum on the CBA, whereas the other AE antibodies were negative. As such, the patient was diagnosed with AE, while PNS was suspected.

The patient was treated with steroids, immunoglobulin, and antiepileptics, achieving an improvement in facial convulsions, and was discharged after steroid dosage adjustment. However, on the 100th day, the symptoms recurred and worsened, with novel presentations of unstable walking, slurred speech, choking during drinking, intellectual impairment, and splenomegaly. The patient scored 18 on the MMSE and 14 on the MoCA scale. The EEG showed predominantly slow background waves and diffuse slow waves. On MRI, the previous lesions had shrunk with old hemorrhagic foci, but new abnormal signals were found in the bilateral paracingulate gyri (**Figure 2**). The serology results revealed the following: Hb, 85 g/L; RBC, 3.02 × 10^12^/L; Plt, 119 × 10^9^/L; LDH, 1,342 U/L; NSE, 105.3 ng/mL (range: <20 ng/mL); sCD25, 9,861 U/mL; serum ferritin, 2,078.25 ng/mL, and positive EBV DNA. The bone marrow cytology revealed phagocytosis; however, no abnormal lymphocyte was found on biopsy. Anti-CASPR2 was negative in the serum on CBA, and the CSF was only weakly positive by TBA. Therefore, the patient was diagnosed with HLH, suspected to be caused by EBV-positive lymphoma, and was treated with the HLH-2004 protocol of etoposide and dexamethasone.

After 1 month of chemotherapy, no seizures were observed, the symptoms improved, and the abnormal signals decreased on MRI (**Figure 2**). On the 280th day, a bone marrow biopsy revealed lymphocytic infiltration between the bone trabeculae. The results of immunohistochemistry are listed as follows: MPO (myeloid cells +), CD61 (megakaryocytes +), CD71 (erythroid cells +), CD117 and CD34 (few cells +), CD3 (focal T cells +), CD20 and CD19 (+), p53 (approximately 20%, moderately or weakly positive), CD10 (+), c-Myc (approximately 50% +), MUM1 (few cells +), BCL2 (few cells +), BCL6 (+), Ki-67 (approximately 80% +), CD5 (+), and cyclin D1 (few cells +). As assessed by hematologists, this case was considered as B-cell lymphoma stage IVB, but a definitive immunophenotypic diagnosis of the lymphoma type remained unclear. Furthermore, the lesions with foci of malacia decreased in size, and no new lesions were found on MRI (**Figure 2**). Subsequently, R-CHOP was administered; at the final follow-up after treatment with R-CHOP, she scored 22 on the MMSE and 17 on the MoCA, and no lymphoma was found on a bone marrow biopsy.

### 2.3 Case 3

A 36-year-old woman who had undergone myomectomy was admitted to a local hospital for mental and behavioral abnormalities after experiencing a cold, with blurred vision, decreased comprehension, calculation, orientation, memory, and judgment, slowed movements, tense limbs, increased salivation, recurrent fever, and convulsions. On the 4th day, the patient tested positive for anti-N-methyl-D-aspartate receptor IgG (anti-NMDAR, 1:100) and negative on next-generation sequencing (NGS) of the CSF. No significant changes were observed on MRI. Therefore, the patient was diagnosed with AE and was treated with immunoglobulin (20 g/day for 5 days) and methylprednisolone (1,000 mg/day for 5 days, tapered to 250 mg/day). After shock therapy, the EEG result showed diffuse δ waves in the background, while the MRI result showed spots of abnormal signaling in the right radial crown. Anti-NMDAR test (1:100) and NGS were performed as previously described.

The patient was transferred to our hospital on the 17th day after onset. A physical examination revealed the following: T, 38.4°C; P, 90 times/min; R, 26 times/min; and BP 147/75 mmHg. Furthermore, she was observed to exhibit involuntary twitching of the corners of her mouth, increased muscle tone, involuntary movements of the extremities, and stiff neck. A serological analysis revealed the following: WBC, 10.83 × 10^9^/L; pH, 7.419 (range: 7.35–7.45); partial pressure of carbon dioxide (PaCO_2_), 48.5 mmHg (range: 35–45 mmHg); base excess (BE), 6.3 mmol/L (range: 0 ± 2.3 mmol/L); fibrinogen, 1.90 g/L (range: 2.00–4.00 g/L); and D-dimer, 1.37 mg/L (range: 0–0.5 mg/L). Only *Staphylococcus epidermidis* was detected in blood cultures. No significant abnormalities were found in other examinations, including in the liver, kidney, lactic acid, infection, or tumors. Urine analysis revealed 0.5 g/L of protein (range: 0–0.1 g/L), 2 mmol/L ketone bodies (range: 2.0–4.0 mg/L), and 100/μL of RBC (range: 0–5/μL). However, the EEG was severely abnormal, and chest CT showed bilateral pneumonia.

The patient was treated with antiepileptics, anti-infection agents, steroids, immunoglobulin, immunoadsorption (five times), rituximab (twice on the 22nd and 29th days), and electrolyte maintenance. A lumbar puncture was performed on the 33rd day, with subsequent CSF analysis revealing the following: 1 × 10^6^/L WBC, 8 × 10^6^/L RBC, 100 mg/L proteins, 3.28 mmol/L glucose, and positive TBA results. The MRI showed no change. Electromyography (EMG) showed rare F-waves in the right median and bilateral tibial nerves, while PET-CT showed decreased glucose metabolism in the bilateral occipital lobes, inflammation in the bilateral lower lungs, thickened pleura, and adhesions. The serum examination was repeated a week later, revealing the following: 0.90 × 10^9^/L WBC, 113 g/L Hb, 81 × 10^9^/L PLT, 624 U/L alanine aminotransferase (ALT), 595 UL aspartate aminotransferase (AST), 159 U/L gamma-glutamyltransferase (GGT), 769 U/L LDH, 5.6 umo1/L of total bilirubin, 941.37 g/m of ferritin, 1.14 g/L of fibrinogen, and 5,005.00 U/mL of sCD25. As bone marrow biopsy showed increased T-cells and hemophagocytes, the patient was considered to have HLH and was treated with the etoposide-based HLH-2004 protocol in addition to the hepatoprotective and anti-infection symptomatic treatment. However, the patient soon presented with nasal necrosis and a fungal infection. Although she was treated with antifungal therapy, including vancomycin, cefoperazone sodium, tazobactam sodium, and posaconazole tablets, her condition progressed rapidly, with continuously expanding nasal necrosis and a peak fever of 40°C. Immunoglobulin and recombinant human granulocyte colony-stimulating factor (rhG-CSF) were also ineffective. The duration from onset to death was 2 months.

## 3 Discussion

HLH and AE may share some triggers or causes. HLH is a rare hyperinflammatory state caused by an overactivated and ineffective immune response and is divided into two major categories: primary and secondary. Secondary HLH is characterized by immune hyperactivation and dysregulation caused by various triggers, including infections, autoimmune conditions, and lymphomas (3). Infections and lymphomas may also cause AE. Lymphomas are frequently associated with AE (6), as in cases 1 and 2 who showed encephalitis with antibodies positive in serum and CSF at onset, while B-cell lymphoma was diagnosed at follow-up. Therefore, lymphoma may have been the cause of HLH and AE in cases 1 and 2. AE may also be an autoimmune inflammatory response induced by infection, as in case 3, where no tumor, but an upper respiratory tract infection, was found; this is especially true for infection with viruses, which can induce anti-NMDAR encephalitis (7, 8). The poor outcome of case 3 and the persistence of high titers of antibody before and after treatment may have occurred as a result of infection. Ineffective apoptosis, activated by infection or induced by malignant cells, may lead to a sustained inflammatory response or antigen presentation. If immune pathways are impaired, an uncontrolled increase in T lymphocytes, macrophages, and natural killer (NK) cell activation can result in a fulminant cytokine storm, triggering HLH (9).

In cases 1 and 2, Epstein–Barr virus DNA was detected in the serum. Epstein–Barr virus is commonly associated with lymphoma and has been reported in both AE and HLH (3, 10). EBV causes AE by molecular mimicry or by inducing autoantigen exposure. EBV also causes HLH because EBV-infected CTL and NK cells de-functionalize into large granular lymphocytes and exhibit abnormal proliferation, triggering excessive cytokine release and macrophage activation (11, 12). However, no examination for Epstein–Barr virus (EBV) in the CSF was conducted in cases 1 and 2; therefore, we could not conclusively say whether EBV was the cause.

Moreover, whether the HLH in case 3 could have been caused by rituximab administration remains unknown. Immune checkpoint inhibitors (ICI) can modify the immune balance, potentially triggering an acute episode of HLH (13–16). One analysis of the WHO global database for suspected adverse drug reactions found that ICI were considered as the only suspected drugs in 34 of 38 HLH cases (17). Rituximab was not mentioned in these cases. Studies have suggested that rituximab improves symptoms, particularly in EBV-HLH, because it only affects B cells, thus eliminating EBV in B cells (18). The association between rituximab use and HLH requires further investigation.

**Figure 3:**
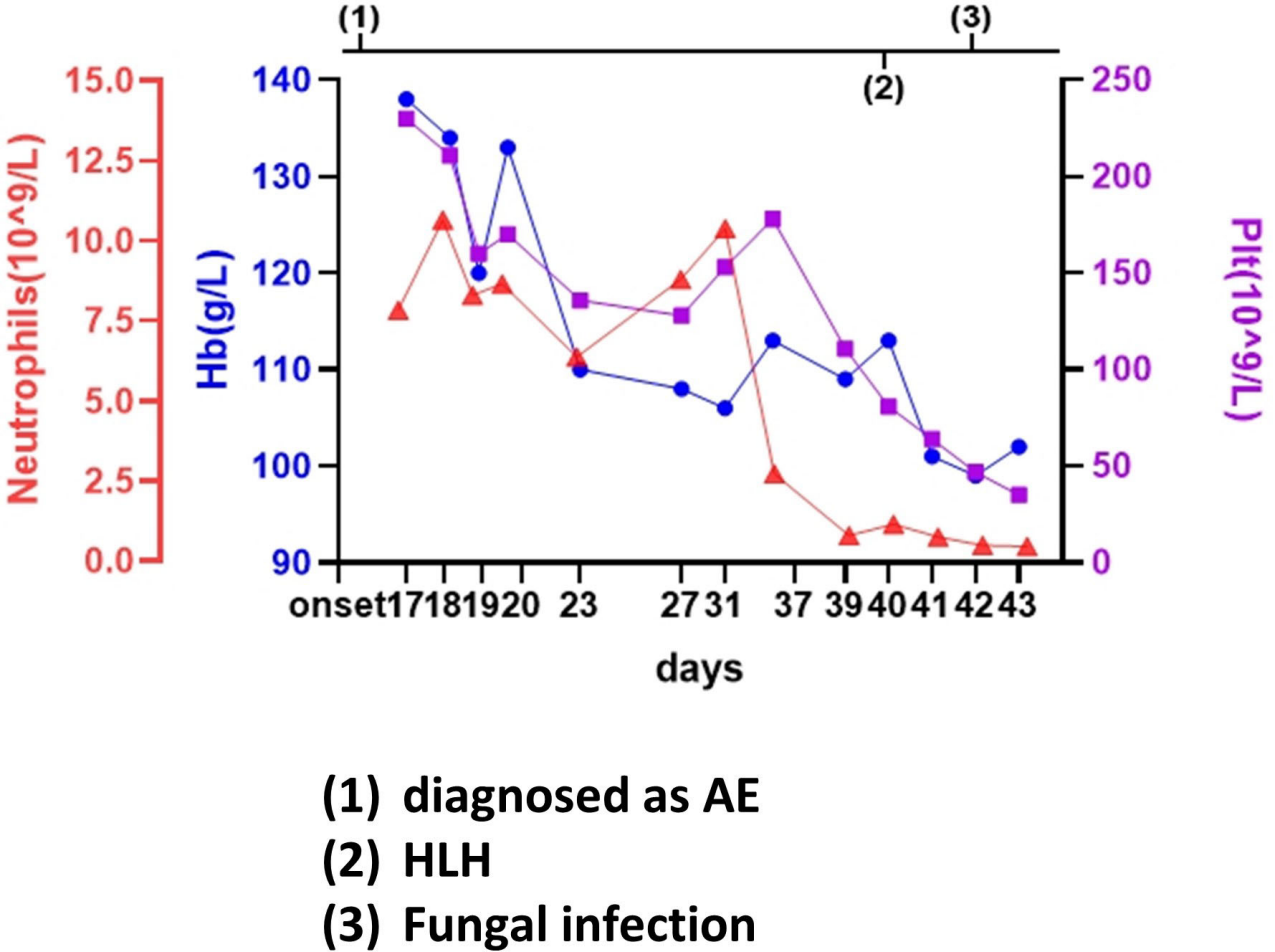
Case 3: A 36-year-old woman experienced hemophagocytic lymphohistiocytosis 36 days after her diagnosis of autoimmune encephalitis. A 36-year-old woman was admitted to our hospital for mental and behavioral abnormality after a cold. Her cerebrospinal fluid tested positive for anti-N-methyl-D-aspartate receptor IgG (anti-NMDAR, 1:100), while it was negative for next-generation sequence (NGS). After the administration of immunoglobulin (20 g/day for 5 days) and methylprednisolone (1,000 mg/day for 5 days, tapered to 250 mg/day to continue), the patient improved a little, and the titer of the antibody remained in the cerebrospinal fluid. Thereafter, she was treated with antiepileptic, anti-infection, steroids, immunoglobulin, immunoadsorption (five times), rituximab (twice, on the 22th and 29th day), and electrolyte maintenance. The counts of platelet, hemoglobin, and neutrophils began to fall on the 37th day. Hemophagocytic lymphohistiocytosis was confirmed on the 40th day. However, the patient soon presented with nasal necrosis, and fungal infection was detected. The details are discussed in the main text.

Whether the early diagnosis should have been CNS HLH at the onset in some of our cases is unclear. For CNS HLH, there were differences between the present cases and previous studies (19–22), including (1) shorter intervals between neurological presentation and systemic HLH; (2) no hemophagocytosis or vacuolated macrophages observed in CSF cytology; (3) no multifocal, bilateral, or symmetric abnormalities on imaging; and (4) relatively stable PLT and Hb at the AE course (**Figures 1**–**3**).

Whether CNS lymphoma was misdiagnosed as AE at the onset also remains unclear. We believe that the diagnosis of AE or secondary CNS lymphoma would have been more appropriate than primary CNS lymphoma based on the following considerations: First, the tumor cells were found in the bone marrow rather than in the CSF. Second, during the administration of immunotherapy, HLH-2004, and R-CHOP, which are not suggested for primary CNS lymphoma, the intracranial lesions shrank (case 2) (23). Third, oligoclonal bands provided a clue as to how lymphoma invaded the central nervous system (CNS). In cases 1 and 2, the oligoclonal bands were type IV, which is different from the specific oligoclonal bands generally observed in AE (type II/III, different bands in the CSF and serum) (24), indicating that the blood–brain barrier was disrupted and antibodies were not intrathecally synthesized. In both cases, peripheral B cell immune responses may have been the cause. Antoine et al. identified the presence of lymphatic vessels between the dura mater and venous sinus connected to the peripheral immune system (25), meaning that the intracranial lesions in the two lymphoma patients may have been caused by the early immune response to lymphoma invading the meninges first along the lymphatic vessels, breaking the blood–brain barrier, and thus entering the brain cortex and subcortex. Above all, AE shares many symptoms with CNS lymphoma, in addition to antibodies and steroid responses; as such, it is difficult to exclude CNS lymphoma without performing brain biopsy (2, 23). However, there was an interesting difference between the present cases. Most importantly, case 1 showed no significant improvement in imaging findings after immunotherapy, whereas in case 2, the lesions decreased in parallel with improved clinical presentations. In the bone marrow biopsy at the time of HLH, lymphoma was found in case 1 but not in case 2. On PET-CT, glucose metabolism was increased in the lesions of case 1 and decreased in those of case 2. Therefore, we considered that secondary CNS lymphoma could have been misdiagnosed as AE in case 1, whereas AE may have been a premonition of potential peripheral lymphoma in case 2. Although CASPR2 antibodies are considered low-risk antibodies for PNS and are associated with malignant thymoma (6), one case of PNS with CASPR2 antibodies and lymphoma has been reported (26). In case 3, who presented with NMDAR encephalitis, hypometabolic changes in the bilateral occipital were seen on PET-CT, which were considered to be associated with NMDAR density in the cerebral cortex (27), so the diagnosis of AE was reasonable.

Different treatment outcomes were observed in the present and previous cases. The previous case improved after immunotherapy without chemotherapy, but the patient remained cognitively impaired due to global cerebral atrophy (4). This case was positive for both anti-NMDAR and α-amino-3-hydroxy-5-methyl-4-isoxazolepropionic acid 1 receptor antibody; as such, it was speculated that the presence of antibodies against two neural surface antigens in this adult patient may have increased the risk of systemic immune activation (4). However, it was chemotherapy, not immunotherapy, that halt HLH in the present study.

Patients should have benefitted from early intrathecal methotrexate. In the present study, methotrexate was not administrated earlier, as the symptoms improved after HLH-2004 and R-CHOP. In case 1, although the symptoms improved, the lesions on MRI did not shrink but instead enlarged later. According to previous studies, intrathecal methotrexate should be prescribed for patients with lymphoma-associated HLH if neurological symptoms persist for 2 to 3 weeks following the initiation of systemic treatment (28, 29). Therefore, case 1 may have benefited from early intrathecal methotrexate therapy.

What are the HLH signals in patients with AE? In a previous study, sudden leukopenia and anemia suggested a bone marrow biopsy in which hemophagocytosis was identified (4). However, in the present study, the neutrophil count did not fall below the standard of HLH (<1.0 × 10^9^/L) over a short time, while PLT decreased suddenly in three cases, and Hb decreased significantly in two cases. As such, we suggest repeating the complete blood count to detect HLH in patients with AE, especially those who improve slightly after immunotherapy or have a risk of lymphoma.

## 4 Conclusion

AE may be a sign of tumors or other diseases; therefore, an active search for its etiology is necessary. Furthermore, it should be noted that AE may be followed by HLH within 1–3 months owing to tumors or infections. Prompt attention should be paid to the decline in blood cells as well as sCD25 and serum ferritin levels, which are important diagnostic indicators of HLH. A pathological examination of the bone marrow is also required to confirm phagocytosis, while early intervention may improve prognosis.

## Data availability statement

The datasets presented in this article are not readily available because this is a case report.

## Ethics statement

The studies involving humans were approved by the Ethics Committee of the Second Affiliated Hospital of Guangzhou Medical University, China. The studies were conducted in accordance with the local legislation and institutional requirements. The participants provided their written informed consent to participate in this study. Written informed consent was obtained from the individual(s) for the publication of any potentially identifiable images or data included in this article.

## Author contributions

LH: Formal analysis, Funding acquisition, Investigation, Methodology, Writing – original draft, Writing – review & editing. JT: Investigation, Visualization, Writing – original draft, Writing – review & editing. PL: Investigation, Visualization, Writing – original draft, Writing – review & editing. ZC: Investigation, Writing – review & editing. QH: Investigation, Writing – review & editing. HY: Investigation, Writing – review & editing. LJ: Investigation, Writing – review & editing. BL: Writing – review & editing. YL: Conceptualization, Formal analysis, Funding acquisition, Investigation, Methodology, Project administration, Supervision, Visualization, Writing – original draft, Writing – review & editing.
